# Supplementary material for: Sphingosine-1-phosphate Decreases Erythrocyte Dysfunction Induced by β-Amyloid
Source: Int J Mol Sci. 2024 May 10;25(10):5184. doi: 10.3390/ijms25105184 (PMC11121638; doi:10.3390/ijms25105184)
Supplement: Supplementary file 1 [file ijms-25-05184-s001.zip › ijms-2941998-supplementary.pdf]

Supplementary material

Inter-assay (as an estimation of the reproducibility) precisions [51]

Table S1A

ATP values (nmoles X 10^8 RBC)

	Mean* (N=5) control	SD*	CV%**	Mean* (N=5) S1P 0,1 mM	SD*	CV%**	Mean* (N=5) S1P 0,5 mM	SD*	CV%**
oxygenated	5,30	0,313	5,90	5,27	0,320	6,08	5,35	0,326	6,09
deoxygenated	17,23	1,034	6,0	16,65	1,047	6,29	19,98	1,209	6,05

Table S1B

ATP values (nmoles X 10^8 RBC)

	Mean* (N=5) control	SD*	CV%**	Mean* (N=5) Abeta	SD*	CV%**	Mean* (N=5) S1P 0,1 mM+ Abeta	SD*	CV%**
oxygenated	5,30	0,427	8,06	5,45	0,436	8,00			
deoxygenated	17,23	1,358	7,88	6,89	0,542	7,86	11,24	0,989	8,80

	Mean* (N=5) S1P 0,5 mM+ Abeta	SD*	CV%**	Mean* (N=5) Abeta+Z-DEVD-FMK	SD*	CV%**
oxygenated						
deoxygenated	17,34	1,542	8,89	17,28	1,289	7,46

Table S2

ATP values (nmoles X 10^8 RBC)

	Mean* N=6	SD*	CV%**
control	17,23	0,791	4,59
Abeta	6,89	0,368	5,34
mastoparan 7	34,45	1,723	5,00
mastoparan 7 + Abeta	35,00	1,778	5,08
S1P 0,1 mM + mastoparan 7 + Abeta	34,36	1,680	4,89

Table S3

cAMP (pmoles 2x 10^5 RBC)

	Mean* N=5	SD*	CV%**
control	9,27	0,753	8,12
S1P 0,1 mM	9,45	0,737	7,80
S1P 0,5 mM	12,34	1,093	8,86

Table S3

cAMP (pmoles 2x 10^5 RBC)

	Mean* N=5	SD*	CV%**
control	9,27	0,753	8,12
Abeta	5,32	0,415	7,80
S1P 0,1 mM+ Abeta	9,10	0,678	7,45
S1P 0,5 mM+ Abeta	12,98	1,038	8,00

Table S4

2,3 DPG mmol l-1

	Mean* N=5	SD*	CV%**
control	*4,9*	0,314	6,40
S1P 0,1 mM	5,2	0,291	5,60
S1P 0,5 mM	5,9	0,368	6,23
S1P 0,5 mM + Abeta	4,5	0,275	6,10
Z-DEVD-FMK + Abeta 0,1 mM	4,8	0,283	5,89

Table S5

Abs 405 nm (a.u.)

	Mean* 12 h N=5	SD*	CV%**	Mean* 24 h N=5	SD*	CV%**
control	0,0390117	0,0020	5,22	0,0492	0,0022	4,56
Abeta	0,098982789	0,0048	4,85	0,13465	0,0066	4,89
S1P 0,5 mM	0,0360117	0,0016	4,50	0,044	0,0022	5,00
Abeta+ S1P 0,5 mM	0,055	0,0031	5,65	0,064	0,0036	5,60
Abeta+Z-DEVD-FMK	0,0079197	0,0004	5,00	0,024	0,0012	4,98
Abeta+ S1P 0,1 mM	0,08	0,0038	4,76	0,09	0,0043	4,74
Mastoparan 7	0,043	0,0021	4,89	0,048	0,0021	4,30

*Mean ± standard deviation of results obtained by separate experiments on different days.

**Precision is the coefficient of variance (CV) obtained by dividing the standard deviation (SD) of all the measurements by their mean (CV% = SD × 100/mean).

References

51. Mossoro-Kpinde, C.D., Kouabosso, A., Mboumba Bouassa, RS. *et al.* Performance evaluation of the touchscreen-based Muse™ Auto CD4/CD4% single-platform system for CD4 T cell numeration in absolute number and in percentage using blood samples from children and adult patients living in the Central African Republic. *J Transl Med* **14**, 326 (2016). https://doi.org/10.1186/s12967-016-1082-7.
